# Supplementary material for: Disturbing the Redox Balance Using Buthionine Sulfoximine Radiosensitized Somatostatin Receptor-2 Expressing Pre-Clinical Models to Peptide Receptor Radionuclide Therapy with 177Lu-DOTATATE
Source: Cancers (Basel). 2023 Apr 17;15(8):2332. doi: 10.3390/cancers15082332 (PMC10137255; doi:10.3390/cancers15082332)
Supplement: Supplementary file 1 [file cancers-15-02332-s001.zip › cancers-2290768-supplementary.pptx]

## Slide 1
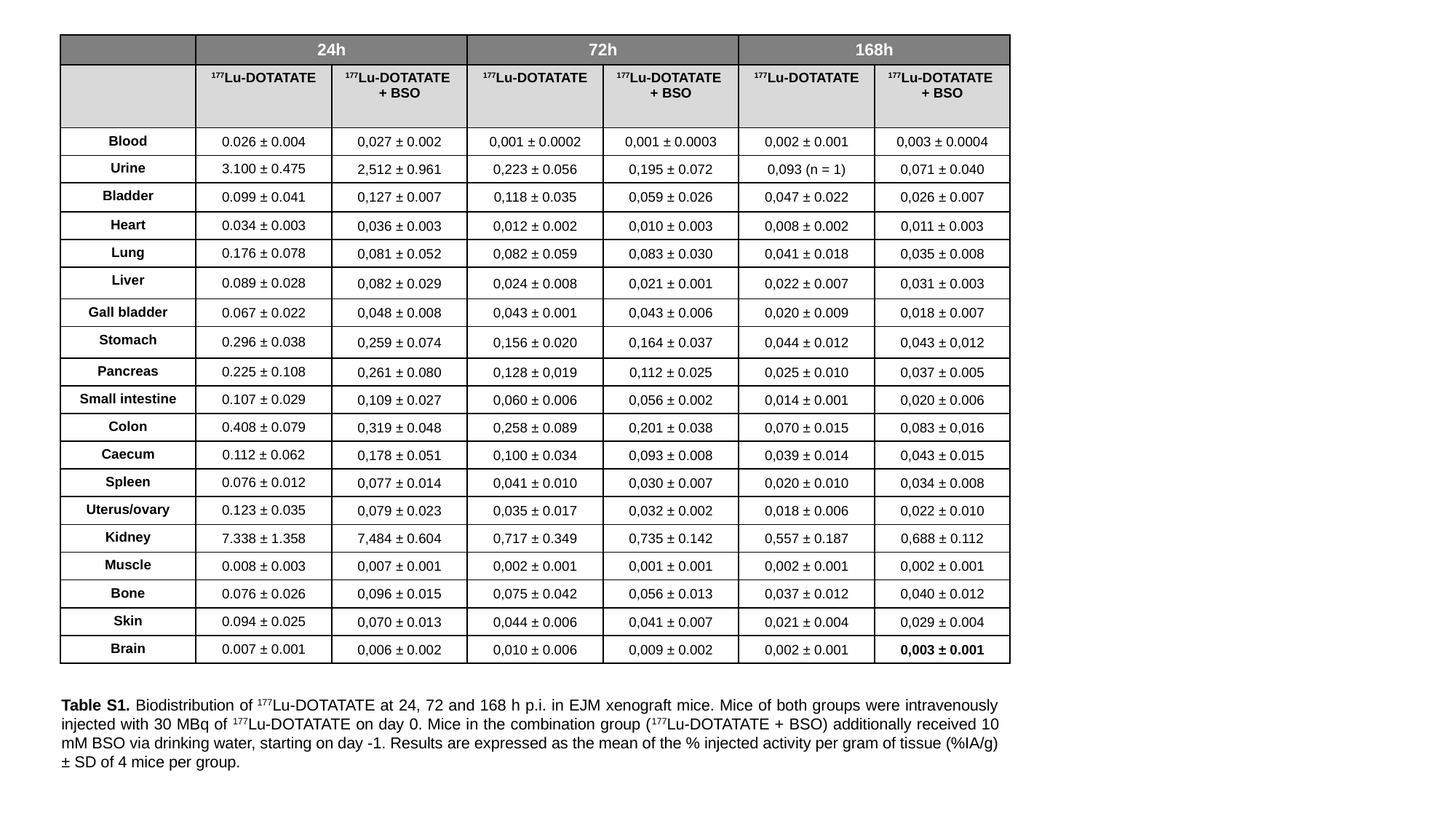

| | 24h | | 72h | | 168h | |
| --- | --- | --- | --- | --- | --- | --- |
| | 177Lu-DOTATATE | 177Lu-DOTATATE + BSO | 177Lu-DOTATATE | 177Lu-DOTATATE + BSO | 177Lu-DOTATATE | 177Lu-DOTATATE + BSO |
| Blood | 0.026 ± 0.004 | 0,027 ± 0.002 | 0,001 ± 0.0002 | 0,001 ± 0.0003 | 0,002 ± 0.001 | 0,003 ± 0.0004 |
| Urine | 3.100 ± 0.475 | 2,512 ± 0.961 | 0,223 ± 0.056 | 0,195 ± 0.072 | 0,093 (n = 1) | 0,071 ± 0.040 |
| Bladder | 0.099 ± 0.041 | 0,127 ± 0.007 | 0,118 ± 0.035 | 0,059 ± 0.026 | 0,047 ± 0.022 | 0,026 ± 0.007 |
| Heart | 0.034 ± 0.003 | 0,036 ± 0.003 | 0,012 ± 0.002 | 0,010 ± 0.003 | 0,008 ± 0.002 | 0,011 ± 0.003 |
| Lung | 0.176 ± 0.078 | 0,081 ± 0.052 | 0,082 ± 0.059 | 0,083 ± 0.030 | 0,041 ± 0.018 | 0,035 ± 0.008 |
| Liver | 0.089 ± 0.028 | 0,082 ± 0.029 | 0,024 ± 0.008 | 0,021 ± 0.001 | 0,022 ± 0.007 | 0,031 ± 0.003 |
| Gall bladder | 0.067 ± 0.022 | 0,048 ± 0.008 | 0,043 ± 0.001 | 0,043 ± 0.006 | 0,020 ± 0.009 | 0,018 ± 0.007 |
| Stomach | 0.296 ± 0.038 | 0,259 ± 0.074 | 0,156 ± 0.020 | 0,164 ± 0.037 | 0,044 ± 0.012 | 0,043 ± 0,012 |
| Pancreas | 0.225 ± 0.108 | 0,261 ± 0.080 | 0,128 ± 0,019 | 0,112 ± 0.025 | 0,025 ± 0.010 | 0,037 ± 0.005 |
| Small intestine | 0.107 ± 0.029 | 0,109 ± 0.027 | 0,060 ± 0.006 | 0,056 ± 0.002 | 0,014 ± 0.001 | 0,020 ± 0.006 |
| Colon | 0.408 ± 0.079 | 0,319 ± 0.048 | 0,258 ± 0.089 | 0,201 ± 0.038 | 0,070 ± 0.015 | 0,083 ± 0,016 |
| Caecum | 0.112 ± 0.062 | 0,178 ± 0.051 | 0,100 ± 0.034 | 0,093 ± 0.008 | 0,039 ± 0.014 | 0,043 ± 0.015 |
| Spleen | 0.076 ± 0.012 | 0,077 ± 0.014 | 0,041 ± 0.010 | 0,030 ± 0.007 | 0,020 ± 0.010 | 0,034 ± 0.008 |
| Uterus/ovary | 0.123 ± 0.035 | 0,079 ± 0.023 | 0,035 ± 0.017 | 0,032 ± 0.002 | 0,018 ± 0.006 | 0,022 ± 0.010 |
| Kidney | 7.338 ± 1.358 | 7,484 ± 0.604 | 0,717 ± 0.349 | 0,735 ± 0.142 | 0,557 ± 0.187 | 0,688 ± 0.112 |
| Muscle | 0.008 ± 0.003 | 0,007 ± 0.001 | 0,002 ± 0.001 | 0,001 ± 0.001 | 0,002 ± 0.001 | 0,002 ± 0.001 |
| Bone | 0.076 ± 0.026 | 0,096 ± 0.015 | 0,075 ± 0.042 | 0,056 ± 0.013 | 0,037 ± 0.012 | 0,040 ± 0.012 |
| Skin | 0.094 ± 0.025 | 0,070 ± 0.013 | 0,044 ± 0.006 | 0,041 ± 0.007 | 0,021 ± 0.004 | 0,029 ± 0.004 |
| Brain | 0.007 ± 0.001 | 0,006 ± 0.002 | 0,010 ± 0.006 | 0,009 ± 0.002 | 0,002 ± 0.001 | 0,003 ± 0.001 |
Table S1. Biodistribution of 177Lu-DOTATATE at 24, 72 and 168 h p.i. in EJM xenograft mice. Mice of both groups were intravenously injected with 30 MBq of 177Lu-DOTATATE on day 0. Mice in the combination group (177Lu-DOTATATE + BSO) additionally received 10 mM BSO via drinking water, starting on day -1. Results are expressed as the mean of the % injected activity per gram of tissue (%IA/g) ± SD of 4 mice per group.

## Slide 2
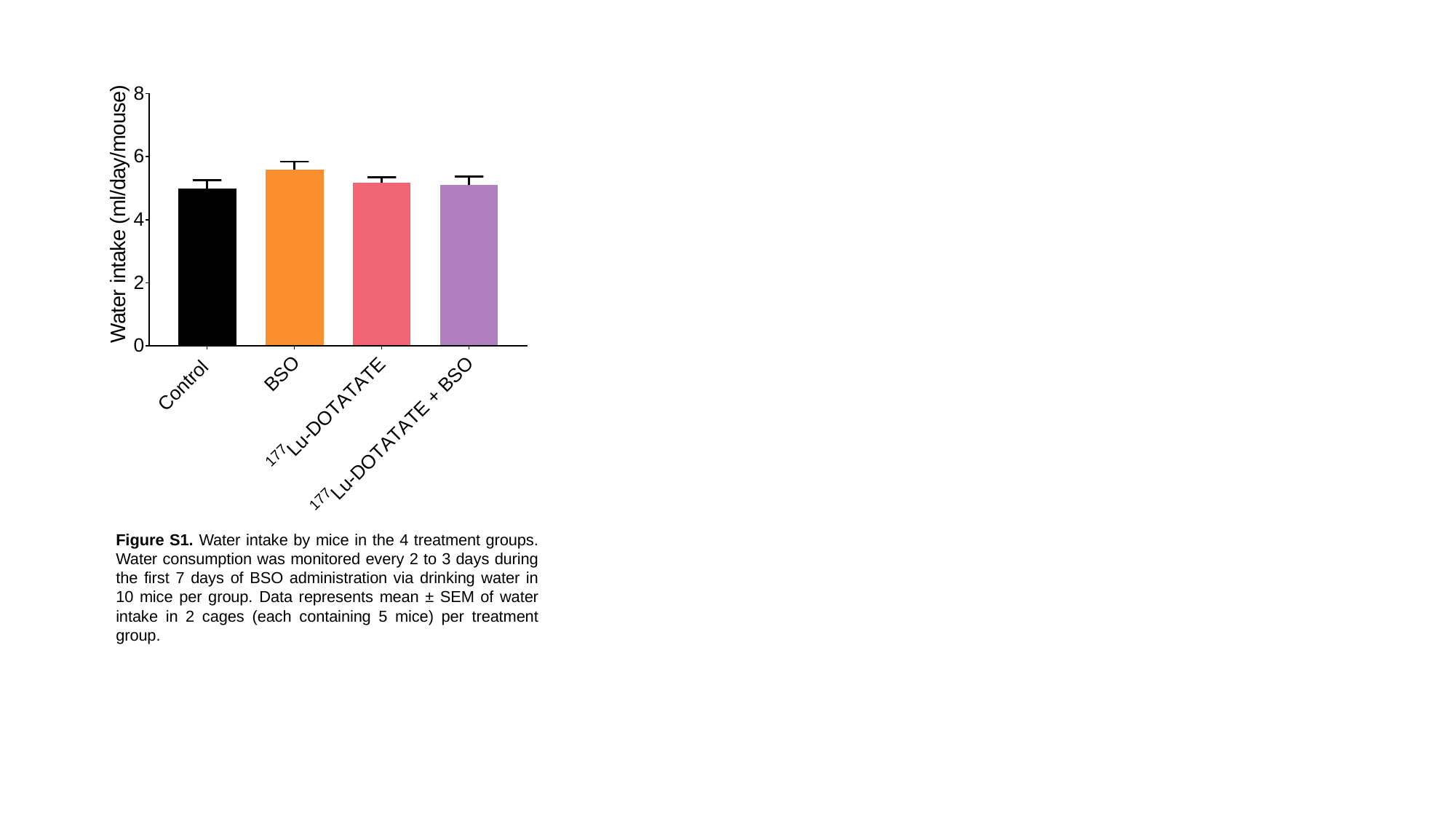

Figure S1. Water intake by mice in the 4 treatment groups. Water consumption was monitored every 2 to 3 days during the first 7 days of BSO administration via drinking water in 10 mice per group. Data represents mean ± SEM of water intake in 2 cages (each containing 5 mice) per treatment group.
